# Rational strategies for designing next-generation oncolytic viruses based on transcriptome analysis of tumor cells infected with oncolytic herpes simplex virus-1

**DOI:** 10.3389/fonc.2024.1469511

**Published:** 2025-01-09

**Authors:** Naeme Javid, Shahriyar Abdoli, Majid Shahbazi

**Affiliations:** ^1^ Department of Molecular Medicine, School of Advanced Technologies in Medicine, Golestan University of Medical Sciences, Gorgan, Iran; ^2^ Department of Medical Biotechnology, School of Advanced Technologies in Medicine, Golestan University of Medical Sciences, Gorgan, Iran; ^3^ Medical Cellular and Molecular Research Center, Golestan University of Medical Sciences, Gorgan, Iran; ^4^ AryaTina Gene (ATG) Biopharmaceutical Company Gorgan, Gorgan, Iran

**Keywords:** oncolytic, HSV-1, transcriptome, hub gene, GADD45g

## Abstract

**Introduction:**

Oncolytic herpes simplex viruses (oHSVs) are a type of biotherapeutic utilized in cancer therapy due to their ability to selectively infect and destroy tumor cells without harming healthy cells. We sought to investigate the functional genomic response and altered metabolic pathways of human cancer cells to oHSV-1 infection and to elucidate the influence of these responses on the relationship between the virus and the cancer cells.

**Methods:**

Two datasets containing gene expression profiles of tumor cells infected with oHSV-1 (G207) and non-infected cells from the Gene Expression Omnibus (GEO) database were processed and normalized using the R software. Common differentially expressed genes between datasets were selected to identify hub genes and were further analyzed. Subsequently, the expression of hub genes was verified by real-time polymerase chain reaction (qRT-PCR) in MDA-MB-231 (a breast cancer cell line) infected with oHSV-1 and non-infected cells.

**Results:**

The results of our data analysis indicated notable disparities in the genes associated with the proteasome pathway between infected and non-infected cells. Our ontology analysis revealed that the proteasome-mediated ubiquitin-dependent protein catabolic process was a significant biological process, with a p-value of 5.8E−21. Additionally, extracellular exosomes and protein binding were identified as significant cellular components and molecular functions, respectively. Common hub genes with degree and maximum neighborhood component (MNC) methods, including PSMD2, PSMD4, PSMA2, PSMD14, PSMD11, PSMC3, PSMC2, PSMD8, and PSMA4, were also identified. Analysis of gene expression by qRT-PCR and differential gene expression revealed that GADD45g genes can be effective genes in the proliferation of oncolytic HSV-1 virus.

**Conclusion:**

The transcriptome changes in tumor cells infected by oHSV-1 may be utilized to predict oncolytic efficacy and provide rational strategies for designing next-generation oncolytic viruses.

## Graphical Abstract

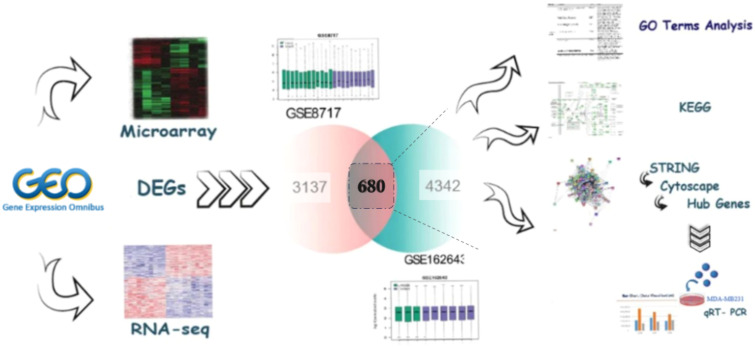

## Introduction

Oncolytic virotherapy represents a novel clinical approach to cancer treatment that utilizes engineered viruses to eradicate cancer cells (1). These viruses are specifically designed to replicate within cancerous tissues while preserving normal tissues and can function as vectors for genes of interest (2).

Imlygic (talimogene laherparepvec or T-VEC) is an oncolytic virus candidate for melanoma treatment, demonstrating promising results in additional cancer types such as colon carcinoma and breast cancer, thus highlighting the substantial potential of oncolytic virotherapy (3). These viruses reduce tumor burden via mechanisms including direct cell lysis (virus replication), induction of antitumor immunity, and disruption of tumor vasculature (4). The findings from clinical trials using oncolytic viruses (OVs) as a treatment indicated its potential importance and significance in future applications and posed challenges in developing OVs as novel weapons for tactical decisions in cancer treatment (5–7).

The recent robust results of oncolytic viruses as a treatment in clinical trials have led to more attention being drawn to further understanding the interactions between the virus and the cancer cell to improve the efficacy of this emerging treatment method. Four key strategies for monitoring oncolytic viruses have been assessed: general gene expression in tumor cells, specific gene expression in tumor cells, transgene expression introduced into the virus, and viral gene expression of particular genes (8–11).

A comprehensive review of multiple studies involving arrays revealed that while herpes simplex virus-1 (HSV-1) infection led to the downregulation of the majority of genes in cells, a larger number of cellular genes were actually upregulated, particularly those involved in regulating the antiviral response and transcriptional regulation. The cellular response to infection is intentional and may be critical for virus propagation (8).

In order to improve therapeutic approaches for combating tumor growth, it is essential to gain a deeper comprehension of the changes that occur following oncolytic virotherapy. Our primary goal was to identify changes in the transcriptome of tumor cells infected with oncolytic HSV-1 (oHSV-1) and to propose methods to improve the virus’s performance. We achieved this by comparing two datasets of infected cells to those of non-infected cells and identifying a comprehensive list of both upregulated and downregulated genes. We conducted ontology analysis, investigated protein–protein interactions, and used real-time polymerase chain reaction (qRT-PCR) to verify gene expression.

## Materials and methods

### Experimental design

**Figure 1:**
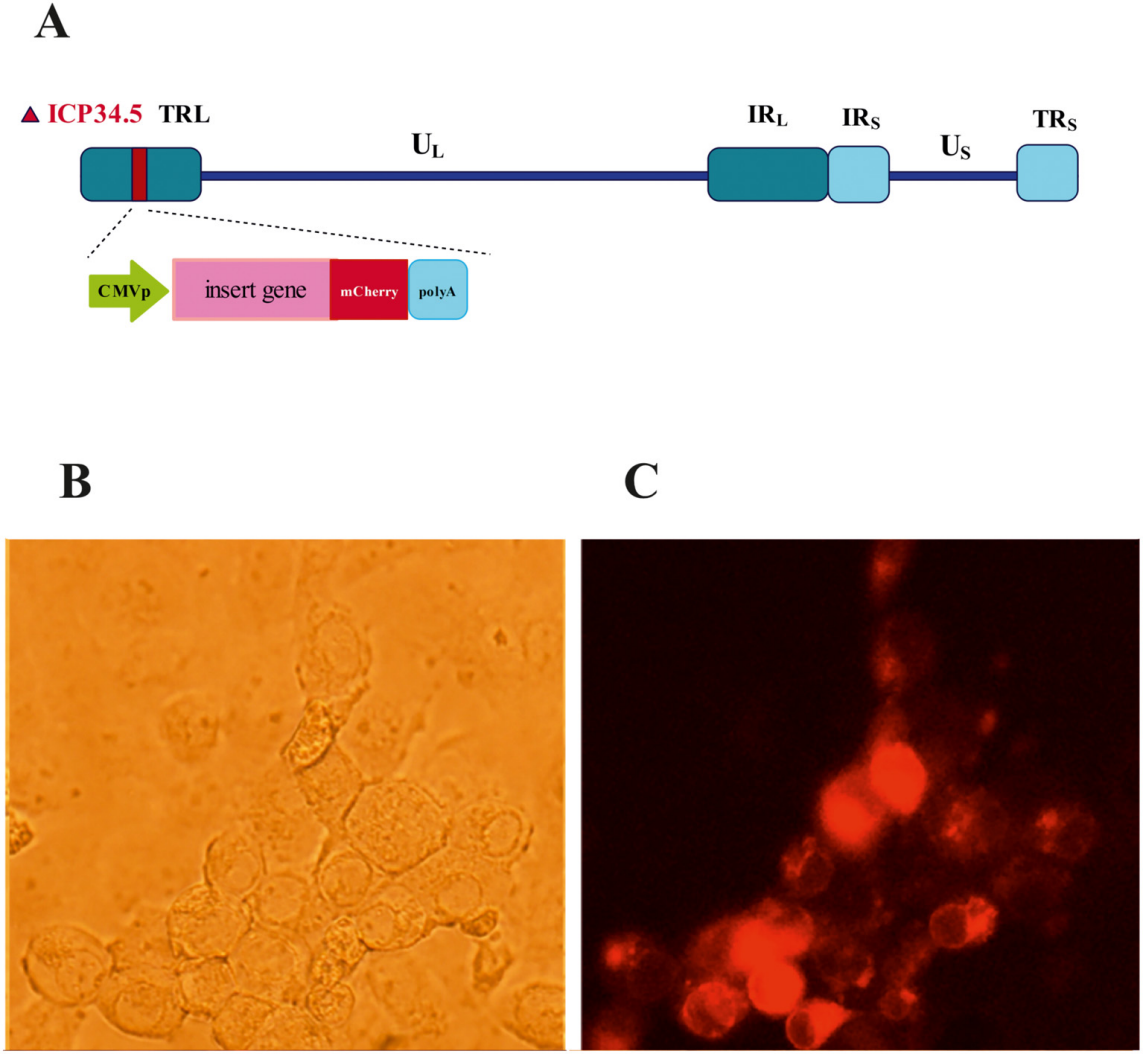
Design and isolation of recombinant oHSV-1 mCherry positive. **(A)** Schematic depiction of the recombinant herpes simplex virus (HSV) genome with its unique long (UL) and unique short (US) regions flanked by inverted repeat elements and deletion in ICP34.5 genes. **(B)** Bright field and **(C)** mCherry under fluorescence microscopy with green filter.

A general diagram of data collection, processing, and analysis in this study is provided in the **Graphical Abstract**. A novel oncolytic HSV-1 virus was engineered, in which the ICP34.5 genes were deleted, and the single-chain antibody as transgene replaced it. This replacement was performed in only one copy of ICP34.5 (**Figure 1A**). The recombinant virus was identified and isolated using mCherry fluorescent dye expression (**Figures 1B, C**). The cytotoxicity of oHSV-1 was confirmed by necessary controls using standard methods. The available datasets containing data from cancer cells infected with oncolytic HSV-1 viruses were initially examined from the available databases (GSE8717 and GSE162643), these datasets were analyzed to compile a comprehensive list of genes with increased and decreased expression, and signaling pathways related to the virus’s functionality were further identified through ontology and Kyoto Encyclopedia of Genes and Genomes (KEGG) pathway analyses. For validation of bioinformatics results from differentially expressed genes (DEGs), nine genes with significantly increased expression were selected. MDA-MB-231 cell line (breast cancer) was infected with our engineered oHSV-1. The supernatants of the infected and non-infected cells were collected, and RNA was extracted. Subsequently, the expression of the selected genes was quantified using real-time PCR.

### Data processing and identification of differentially expressed genes

The crucial gene expression data were meticulously retrieved from the Gene Expression Omnibus (GEO) database (https://www.ncbi.nlm.nih.gov/geo/). The first dataset (GSE8717) submitted by Mahller et al. contained five human malignant peripheral nerve sheath tumor (MPNST) cancer cell lines infected with G207 or mock (5). The second dataset (GSE162643) was submitted by Miller et al. (12). It contained six infected and three mock data. In this study, data were first normalized and then background corrected. The processed data were meticulously screened for DEGs using the limma package, with screening criteria set at adjusted p > 0.01 and log2FC > 2 (fold change).

### Analysis of pathway enrichment

Gene Ontology (GO) analysis and KEGG pathway analysis were conducted using DAVID (http://david.abcc.ncifcrf.gov/), a widely utilized online platform for annotative and functional information associated with extensive gene lists. These analyses were performed with a significance threshold of p < 0.01, indicating a statistically significant difference (13).

### PPI network construction and screening of critical genes

The STRING database (https://www.string-db.org/) enables us to build a protein–protein interaction (PPI) network of target genes. cytoHubba is a simple Cytoscape plugin that uses various algorithms to determine the importance of nodes in a PPI network. Ten genes using the degree algorithm were selected as hub genes.

### Cell culture and oHSV-1 infection

The MDA-MB-231 cells were grown in DMEM media (Gibco, Grand Island, NY, USA) containing 10% fetal bovine serum (FBS) and 1% pen strep. Sub-confluent MDA-MB-231 cells grown in six-well plates were infected with oHSV-1 at a multiplicity of infection (MOI) of 5, and the supernatant was collected 6 hours post-infection.

### Real-time PCR

To extract total RNA, TRIzol reagent was used according to the manufacturer’s instructions. RNA concentration was assessed using a NanoDrop (DeNovix DS-11). RNA (2 µg) was subjected to DNase treatment, and cDNA derived from this RNA was synthesized using the AddScript cDNA Synthesis Kit (Addbio Company, Linkoping, Sweden) according to the manufacturer’s protocol. Subsequently, real-time PCR was performed using the RealQ Plus 2x Master Mix Green High ROX™ (Ampliqon, Odense, Denmark). GAPDH was used as an internal reference control. The primers used to amplify each gene are listed in **Supplementary Table S1** supplementary files. Gene expression levels were determined by calculating ΔΔCt relative quantification, and significant differences in expression levels between infected and uninfected cells were determined based on these measurements. Data analysis was performed using a t-test with GraphPad Prism 9.

## Results

### Identification of a list of differentially expressed genes from datasets

**Figure 2:**
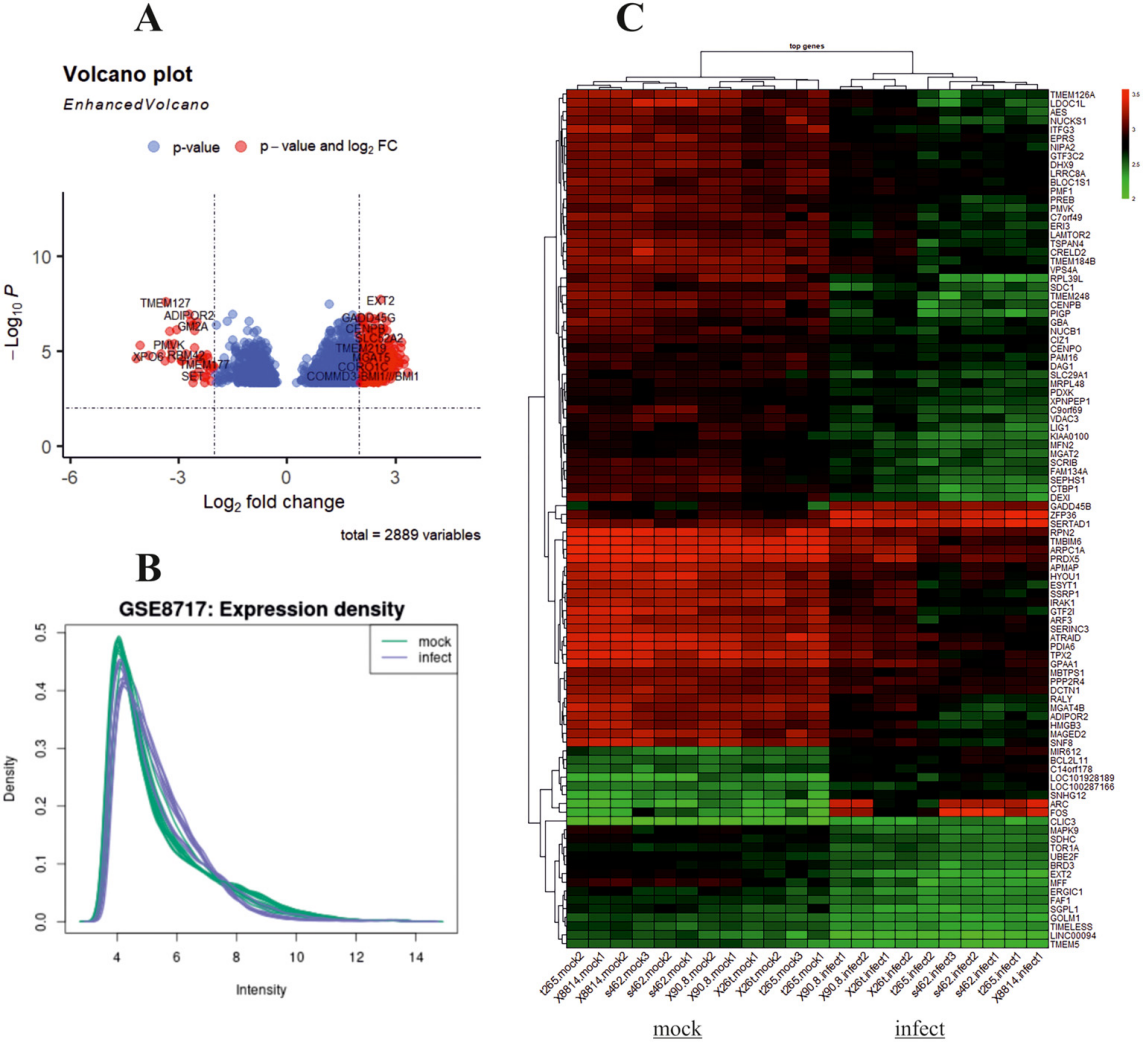
Analysis of GSE8717 using R. Volcano plot, histogram, and heatmap of differentially expressed genes (DEGs) with screening criteria of log2 FC ≥ 2 and adjusted p-value <0.05. **(A)** Volcano plot showing the DEGs in microarray infected compared to uninfected samples. **(B)** Histogram. **(C)** Heatmap.

**Figure 3:**
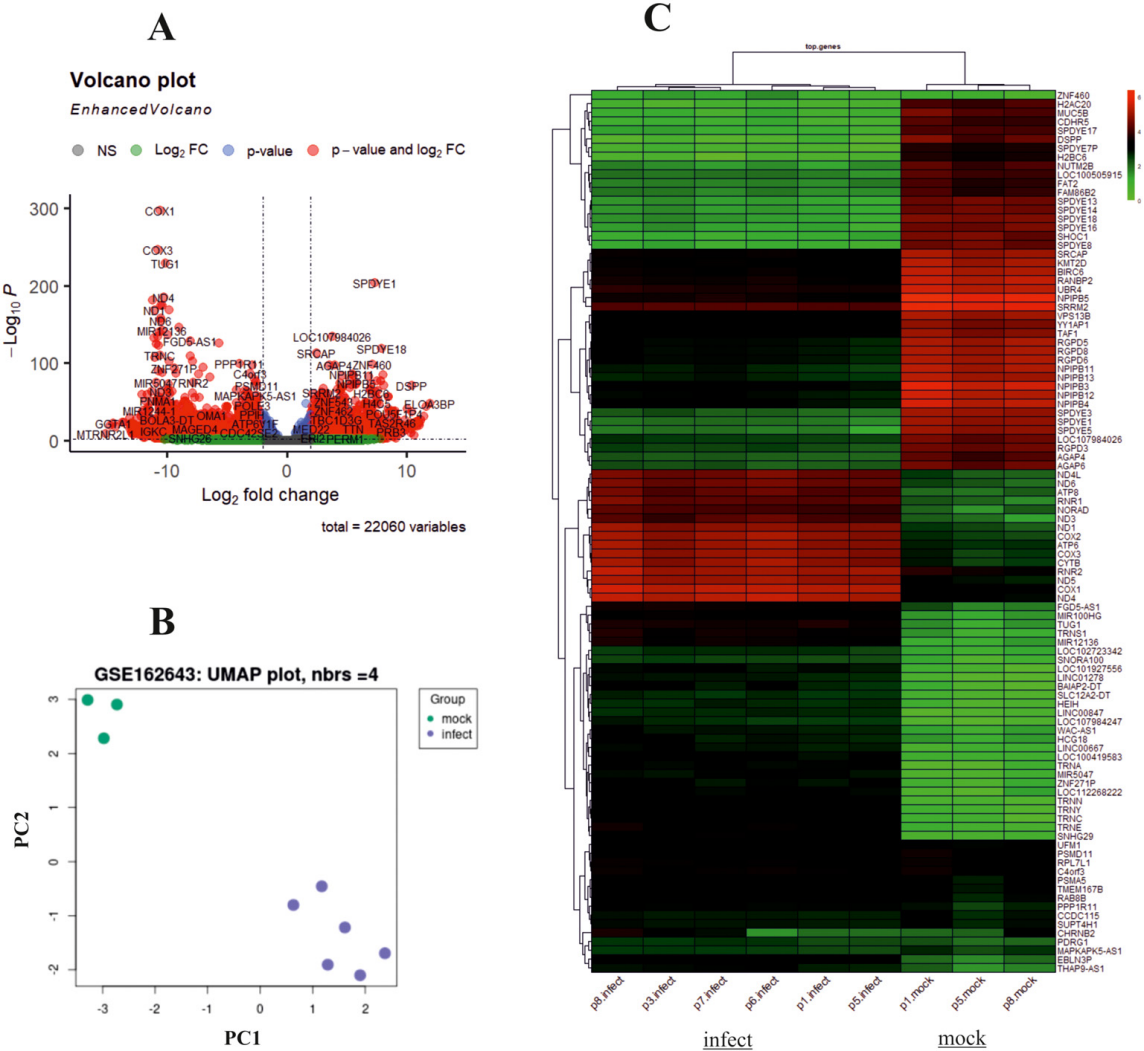
Analysis of GSE162643 using R. Volcano plot, principal component analysis (PCA), and heatmap of differentially expressed genes (DEGs) with screening criteria of log2 FC ≥ 2 and adjusted p-value <0.05. **(A)** Volcano plot showing the DEGs in microarray infected compared to uninfected samples. **(B)** PCA dataset shows suitable quality of microarray samples. **(C)** Heatmap.

**Figure 4:**
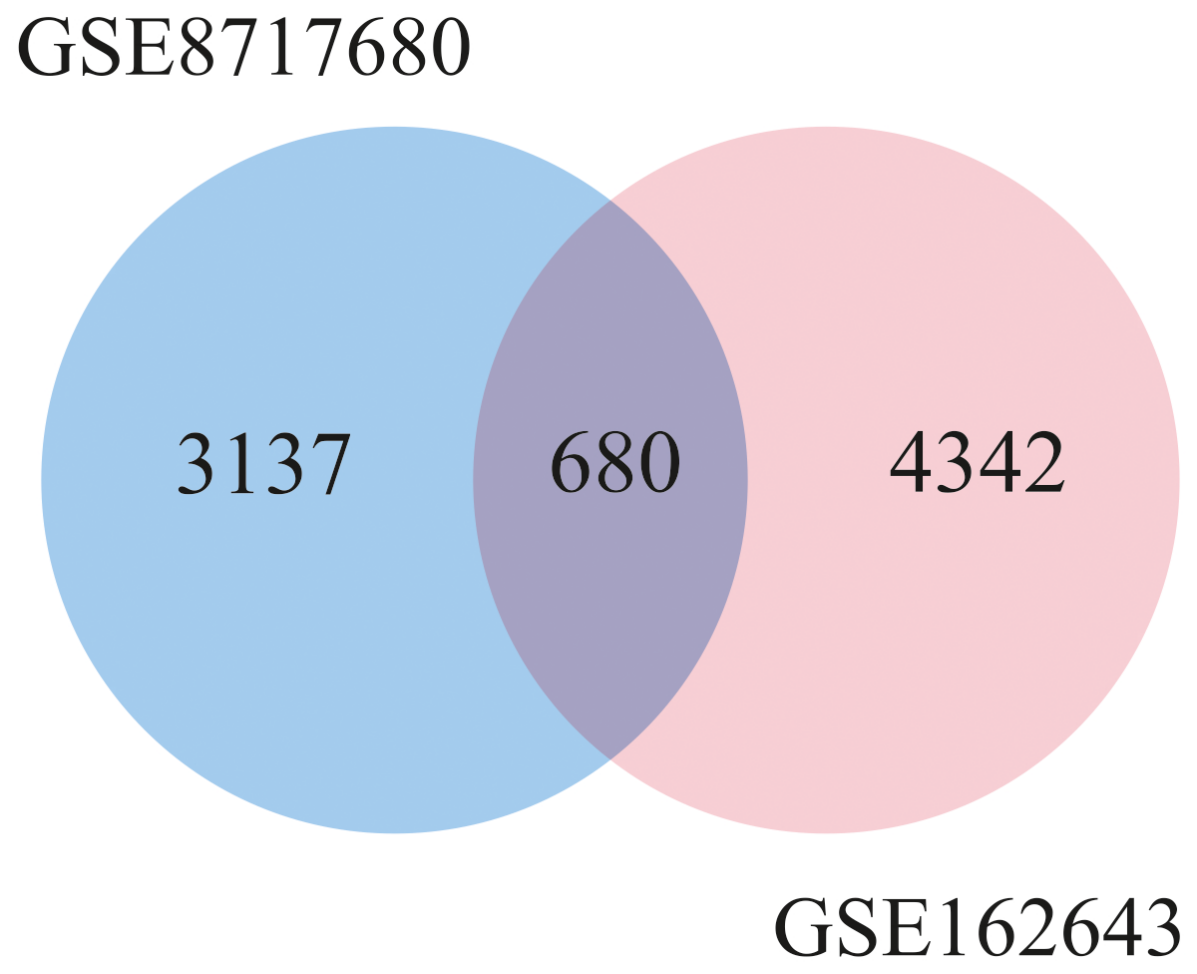
Venn diagram of common differentially expressed genes between two datasets (GSE8717 and GSE162643).

The design of this study is illustrated in the **Graphical Abstract**. To gain some insight into how oHSV-1 affects the expression of genes and to make a better understanding of alteration in affected signaling pathways, two datasets (GSE8717 and GSE162643) were processed and normalized using the R software. The GSE8717 dataset consists of five samples of polymorphonuclear neutrophils (PMNs) infected with G207 oHSV-1 and five samples of uninfected PMNs, which contained 3,874 DEGs (p-value <0.01 and fold change >2), including 3,530 upregulated genes and 344 downregulated genes. The initial analysis of the dataset using histogram, heatmap, and volcano plots is shown in **Figure 2**. The GSE162643 dataset comprises six adult patients with recurrent glioblastoma who were enrolled in a phase Ib clinical trial to evaluate the safety and efficacy of G207 in promoting antitumor responses. The trial also included three samples of uninfected and contained 5,022 DEGs (p-value <0.01 and fold change >2), including 1,582 upregulated genes and 3,440 downregulated genes. The initial analysis of the dataset using principal component analysis (PCA), heatmap, and volcano plot is shown in **Figure 3**. Volcano plots were utilized to represent variance in DEGs. The DEGs exhibiting high- and low-fold changes were positioned at the top-left and top-right corners, respectively. Furthermore, the top 30 significant genes are demonstrated in **Supplementary Tables S2, S3** for GSE8717 and GSE162643, respectively. Common DEGs between two datasets were investigated. Finally, 680 common genes were found between these two datasets (**Figure 4**).

### Ontology analysis and cellular processes

The GO analysis, a comprehensive and meticulous process, was conducted using DAVID. GO covers three categories, namely, cellular component (CC), biological process (BP), and molecular function (MF) (**Table 1**). One hundred significantly enriched BP terms were found, with the most significant ones being proteasome-mediated ubiquitin-dependent protein catabolic process, mRNA splicing, via spliceosome, and intracellular protein transport. Forty-seven significant CC terms were identified, with the most significant ones being extracellular exosome, proteasome complex, cytosol, proteasome accessory complex, and nucleoplasm. The significantly enriched MF terms were GDP binding, GTPase activity, GTP binding, RNA binding, and Proteasome activating ATPase activity. KEGG pathway enrichment analysis revealed the association of the DEGs including Proteasome, Pathways of neurodegeneration, Spinocerebellar ataxia, Oxidative phosphorylation, and endocytosis (**Table 2**).

**Table 1:** Top 6 Gene Ontology (GO) term analysis revealed enrichment in relevant biological process (BP), cellular component (CC), and molecular function (MF).

Category	GO terms	p-Value	Genes
Biological process	Proteasome-mediated ubiquitin-dependent protein catabolic process	1.8E−13	PCNP, RAD23B, SKP1, SH3BGRL, TNFAIP1, NAKRD9, PEX10, PSMA2, PSMA4, PSMA5, PSMA7, PSMB1, PSMB3, PSMB7, PSMD2, PSMD4, PSMC2, PSMC3, PSMC4, PSMC5, PSMC6, PSMD11, PSMD13, PSMD14, PSMD6, PSMD7, PSMD8, PSMF1, PPP2CB, RNF4, UBE2A, UBE2G1, UBE2H, USP14
	Vesicle-mediated transport	2.2E−7	ARF3, ARF5, ARL1, KXD1, NAPA, RAB10, RAB11A, RAB14, RAB1A, RAB29, RAB2A, RAB2B, SFT2D2, SFT2D3, TBC1D20, AP1S1, AP3M1, CNIH1, JAGN1, MCFD2, PRKCI, SAR1B, STX16, STX7
	mRNA splicing, via spliceosome	3.1E−7	BUD31, LSM3, LSM4, RBM8A, HNRNPA3, HNRNPK, NCBP2, PPIL1, PPIL3, PNN, PRPF19, PRPF31, PRPF40A, PRPF4, PPP1R7, SRSF1, SRSF8, SNU13, SNRPD1, SNRPD3, SNRPF, SF3B5, UBL5
	Intracellular protein transport	3.2E−6	ARF3, ARF5, ARFIP2, ARL1, BCAP31, COPZ1, NAPA, RAB14, RAB18, RAB1A, RAB1B, RAB21, RAB22A, RAB29, RAB31, RAB5A, RAB5B, RAB5C, AP1S1, AP3M1, SAR1B, STX16, STX7, TIMM17A, TMED10, TMED5
	Protein transport	3.4E−6	ARF3, ARF5, ARFGAP2, ARL6IP5, BET1L, CMTM6, COPZ1, ENY2, GABARAPL2, GABARAP, RAB2A, RAB2B, RAB7A, RAN, S100A13, SFT2D2, SFT2D3, AAGAB, CETN2, CHMP2A, CHMP5, GOLT1B, IER3IP1, JAGN1, LMAN2L, MCFD2, PSEN1, RRBP1, SCAMP4, SNX12, SNX3, SERP1, TIMM10B, TMED5, TMEM9
	Synaptic vesicle lumen acidification	8.5E−6	ATP6V0D1, ATP6V1A, ATP6V1B2, ATP6V1C1, ATP6V1E1, ATP6V1G1, ATP6AP2
Cellular component	Extracellular exosome	1.2E−23	ATIC, ARF3, ARF5, ARL15, ATP6V0D1, ATP6V1A, ATP6V1B2, ATP6V1C1, ATP6V1E1, ATP6V1G1, ATP6AP2, ATP1A1, ATP1B3, CD59, CD81, COPS8, FAT1, GNAI3, GNB1, GNG12, GNG2, MOB1A, NAA50, NEDD8, NME1, NRAS, NAPA, PTTG1IP, PARK7, RAB10, RAB11A, RAB14, RAB1A, RAB1B, RAB21, etc.
	Proteasome complex	6.4E−23	RAD23B, PSMA2, PSMA4, PSMA5, PSMA7, PSMB1, PSMB3, PSMB7, PSMD2, PSMD4, PSMC2, PSMC3, PSMC4, PSMC5, PSMC6, PSMD10, PSMD11, PSMD13, PSMD14, PSMD5, PSMD6, PSMD7, PSMD8, PSME3, PSMF1, USP14
	Cytosol	1.5E−15	ADO, ATIC, ARF3, ARFGAP2, ARFIP2, ARL1, ARL2BP, ARL6IP1, ATP6V1A, ATP6V1B2, ATP6V1C1, ATP6V1E1, ATP6V1G1, BCAP31, BANF1, BAG5, BCL2L1, BID, BET1L, CNBP, CNOT1, CNOT8, CD2BP2, COMMD4, COPS6, COPS8, COPZ1, DCAF7, DNAJC5, etc.
	Proteasome accessory complex	3.4E−15	PSMD2, PSMD4, PSMC2, PSMC3, PSMC4, PSMC5, PSMC6, PSMD11, PSMD13, PSMD14, PSMD5, PSMD6, PSMD8
	Nucleoplasm	9.1E−14	ARL2BP, ARL2BP, ATP6V1A, BANF1, BCOR, BUD31, C1D, CD2BP2, CGGBP1, COMMD10, COPS6, COPS8, CTDSP2, CELF1, DCAF7, DDA1, POLE3, DNAJC8, ENY2, EID1, FAM20B, GNAI3, GPR107, GRSF1, HIGD1A, IMP3, IMP4, INO80E, LDOC1, LSM3, LSM4, LYRM1, MRFAP1, etc.
	Mitochondrion	2.4E−13	AGPAT5, AGPAT5, BAG5, BNIP3L, BCL2L1, BID, BRI3BP, CMC2, POLDIP2, DNAJC5, ENY2, FIBP, FUNDC2, GRSF1, HIGD1A, NACC2, NAXD, NDUFA1, NDUFA8, NDUFAB1, NDUFB6, NDFIP2, PET100, PYURF, PARK7, RAB29, RAB7A, RALA, TRIAP1, AK3, ADH5, ARMC1, BSG, BECN1, BLOC1S2, BCAT1, C1orf43, C19orf12, CHCHD2, CHCHD7, etc.
Molecular function	Protein binding	1.8E−36	AGPAT3, AGPAT5, ADO, HACD3, ARF3, ARF5, ARFIP2, ARL1, ARL15, ARL2BP, ARL6IP1, ARL6IP5, ALG5, ARID1A, ATP6V0D1, ATP6V1A, ATP6V1B2, ATP6V1C1, ATP6V1E1, ATP6V1G1, ATP6AP2, ATP1A1, ATP1B3, BCAP31, BANF1, BAG5, BNIP3L, BCL2L1, BCOR, BID, BLCAP, BRI3BP, BUD31, CMC2, C1D, CNBP, CNOT1, CNOT8, CD2BP2, CD59, CD81, CD99L2, etc.
	GDP binding	2.6E−13	GNAI3,NARS,RAB10,RAB14,RAB18, RAB21,RAB22A,RAB29,RAB2A, RAB31, RAB5A, RAB5B, RAB5C, RAB7A,RAN, RAP1B, RAP2A, RAP2C, RALA,RALB, MRAS
	GTPase activity	2.6E−11	ARF3, ARF5, ARL1, ARL15, GNAI3, GNB1, GNG10, NRAS, RAB10, RAB11A, RAB14, RAB18, RAB1A, RAB1B, RAB21, RAB22A, RAB29, RAB2A, RAB2B, RAB31, RAB5A, RAB5B, RAB5C, RAB7A, RAN, RAP1B, RAP2A, RAP2C, RAP2C, RALB, RAC1, ARHGAP5, CDC42, ENTPD4, MRAS, NTPCR, RHOA, RASD1, RGS4, SAR1B
	GTP binding	2.0E−9	ARF3, ARF5, ARFIP2, ARL1, ARL15, GNAI3, NME1, NRAS, RAB10, RAB11A, RAB14, RAB18, RAB1A, RAB1B, RAB21, RAB22A, RAB29, RAB2A, RAB2B, RAB31, RAB5A, RAB5B, RAB5C, RAB7A, RAN, RAP1B, RAP2A, RAP2C, RALA, RALB, RAC1, ARHGAP5, SRPRB, AK3, CDC42, HSP90AA1, MRAS, RHOA, RASD1, SAR1B
	RNA binding	1.5E−7	ARF3, C1D, CNBP, CNOT1, CNOT8, CELF1, ERH, GRSF1, G3BP1, G3BP2, IMP3, LSM3, LSM4, NME1, RANBP2, RAN, RBBP7, RBM8A, RBPMS, RANGAP1, UHMK1, WDR33, BZW1, CANX, CCT4, CCT6A, CLNS1A, C7orf50, CIRBP, CPNE3, DIDO1, EIF1, EIF1AX, EIF4H, GANAB, GRB2, HSP90AA1, HSPD1, HDGF, HNRNPA0, etc.
	Proteasome activating ATPase activity	1.6E−5	PSMC2, PSMC3, PSMC4, PSMC5, PSMC6

**Table 2:** Kyoto Encyclopedia of Genes and Genomes (KEGG) pathway analysis of differentially expressed genes (p < 0.01) (top 9 pathways).

Category	GO term	p-Value	Genes
KEGG_PATHWAY	Proteasome	5.8E−21	PSMA2, PSMA4, PSMA5, PSMA7, PSMB1, PSMB3, PSMB7, PSMD2, PSMD4, PSMC2, PSMC3, PSMC4, PSMC5, PSMC6, PSMD11, PSMD13, PSMD14, PSMD6, PSMD7, PSMD8, PSMD9, PSME3, PSMF1, POMP
KEGG_PATHWAY	Pathways of neurodegeneration	6.7E−10	BCL2L1, BID, NDUFA1, NDUFA5, NDUFA8, NDUFAB1, NDUFB6, NRAS, PARK7, RAB1A, RAB5A, RAC1, VAPB, BECN1, COX6C, COX7B, CYCS, DERL1, PSEN1, PRNP, PSMA2, PSMA4, PSMA5, PSMA7, PSMB1, PSMB3, PSMB7, PSMD2, PSMD4, PSMC2, PSMC3, PSMC4, PSMC5, PSMC6, PSMD11, PSMD13, PSMD14, PSMD6, PSMD7, PSMD8, PSMD9, SDHB, SOD1, UQCRQ, UQCR11, UBE2G1, UBE2G2, VDAC1, VDAC3
KEGG_PATHWAY	Spinocerebellar ataxia	9.9E−10	BECN1, CYCS, PSMA2, PSMA4, PSMA5, PSMA7, PSMB1, PSMB3, PSMB7, PSMD2, PSMD4, PSMC2, PSMC3, PSMC4, PSMC5, PSMC6, PSMD11, PSMD13, PSMD14, PSMD6, PSMD7, PSMD8, PSMD9, VDAC1, VDAC3
KEGG_PATHWAY	Oxidative phosphorylation	1.5E−5	ATP6V0D1, ATP6V1A, ATP6V1B2, ATP6V1C1, ATP6V1E1, ATP6V1G1, NDUFA1, NDUFA5, NDUFA8, NDUFAB1, NDUFB6, COX6C, COX7B, CYCS, PPA2, SDHB, UQCRQ, UQCR11
KEGG_PATHWAY	Endocytosis	1.0E−4	ARF3, ARF5, ARFGAP2, RAB10, RAB11A, RAB22A, RAB31, RAB5A, RAB5B, RAB5C, RAB7A, ACTR2, ARPC1A, ARPC3, ARPC4, ARPC4, CAPZA2, CDC42, CHMP2A, CHMP5, PRKCI, RHOA, SNX12, SNX3
KEGG_PATHWAY	Nucleotide excision repair	1.4E−4	POLE3, RAD23B, POLR2C, POLR2D, POLR2E, POLR2K, POLR2L, CETN2, GTF2H5, PCNA, RPA3
KEGG_PATHWAY	Spliceosome	1.9E−3	BUD31, LSM3, LSM4, RBM8A, HNRNPA3, HNRNPK, NCBP2, PPIL1, PRPF19, PRPF31, PRPF40A, PRPF4, SRSF1, SRSF8, SNU13, SNRPD1, SNRPD3, SNRPF, SF3B5
KEGG_PATHWAY	Chemical carcinogenesis	2.6E−3	NDUFA1, NDUFA5, NDUFA8, NDUFAB1, NDUFB6, NRAS, RAC1, COX6C, COX7B, GSTO1, GRB2, MGST3, PTPN11, SDHB, SOD1, UQCRQ, UQCR11, VDAC1, VDAC3
KEGG_PATHWAY	Ras signaling pathway	4.7E−3	BCL2L1, GNB1, GNG10, GNG12, GNG2, NRAS, RAB5A, RAB5B, RAB5C, RAP1B, RALA, RALB, RAC1, CDC42, GRB2, MRAS, PTPN11, RALBP1, RHOA

### Network analysis of the DEGs

**Figure 5:**
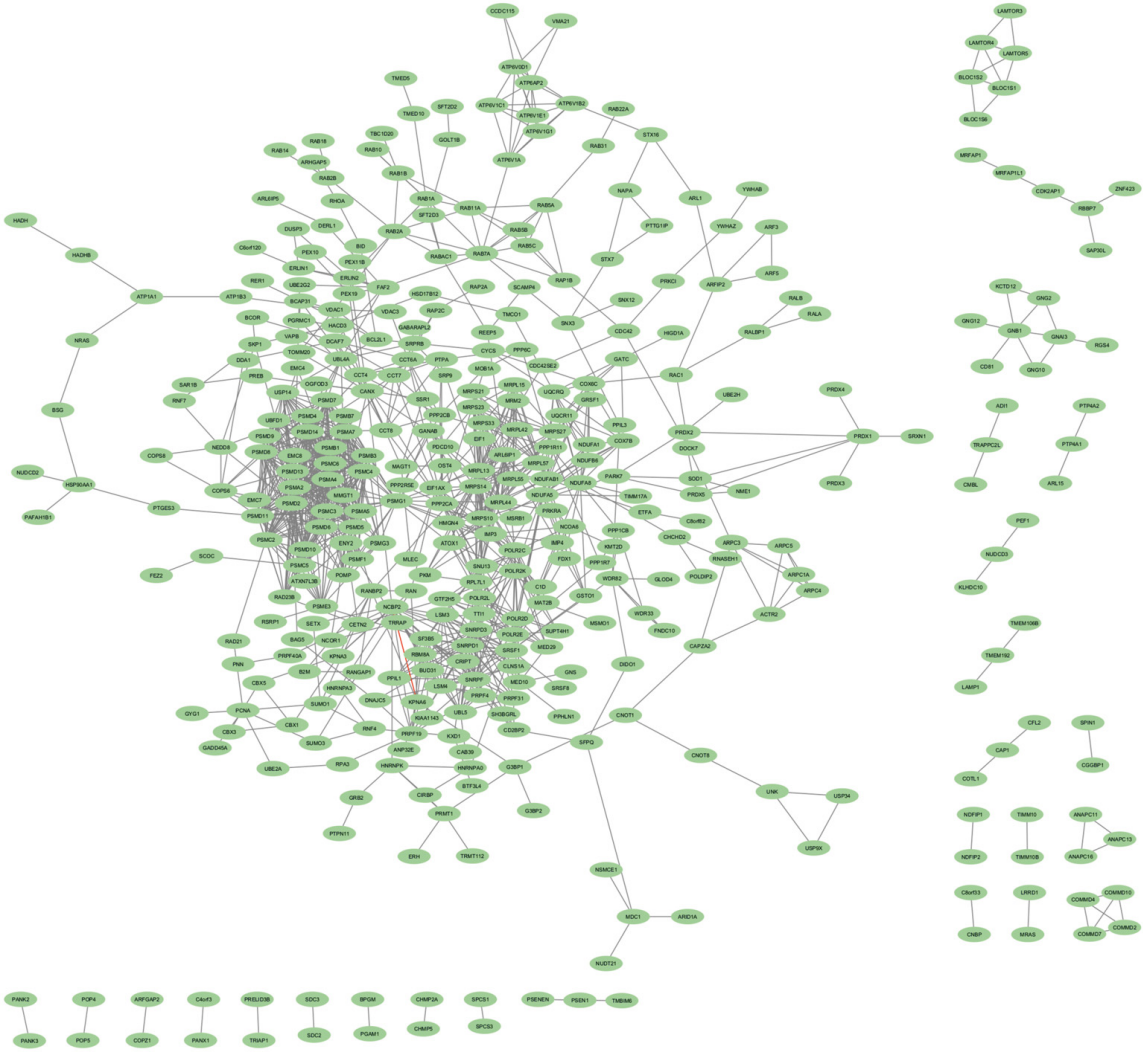
PPI network for identified cDEGs. Nodes indicate cDEGs, and edges represent protein–protein associations. PPI, protein–protein interaction; cDEGs, common differentially expressed genes.

**Figure 6:**
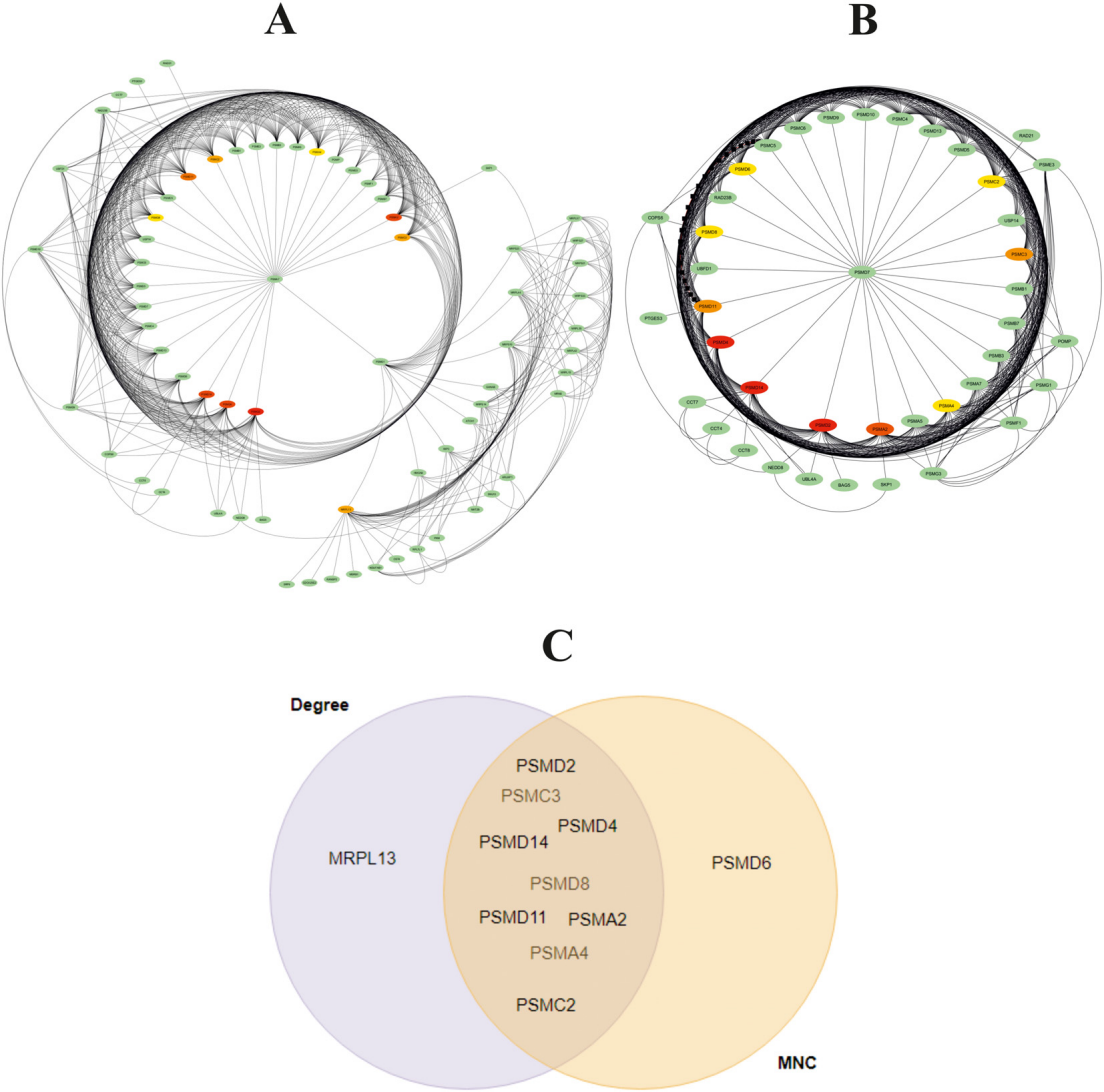
Identification of 10 hub genes using cytoHubba plugin with **(A)** degree and **(B)** maximum neighborhood component (MNC) method in Cytoscape software. The hub gene is marked in red. **(C)** Venn diagram for common hub genes with degree and maximum neighborhood component (MNC) methods.

Cytoscape was used to construct and visualize the PPI networks for DEGs (**Figure 5**). Specifically, the PPI network for DEGs comprised 396 nodes and 2,336 edges, generated using the cytoHubba plugin within the Cytoscape software. The cytoHubba plugin identified 10 genes, namely, PSMD2, PSMD4, PSMA2, PSMD14, PSMD11, PSMC3, PSMC2, MRPL13, PSMC5, and PSMA4, as hub genes that had a more significant influence than the other genes with degree method. The hub genes identified through the degree and maximum neighborhood component (MNC) method are depicted in **Figures 6A, B**, respectively. Common hub genes with two methods are shown in **Figure 6C**.

Network analysis showed that PSMD2 (Proteasome 26S Subunit Ubiquitin Receptor, Non-ATPase 2) has the highest degree (31 degree). Its related pathways are Regulation of activated PAK-2p34 by proteasome-mediated degradation and Assembly of the pre-replicative complex. The GO annotations for this gene comprise binding and enzyme regulator activity.

### Quantitative RT-PCR analysis to confirm differentially expressed genes

**Figure 7:**
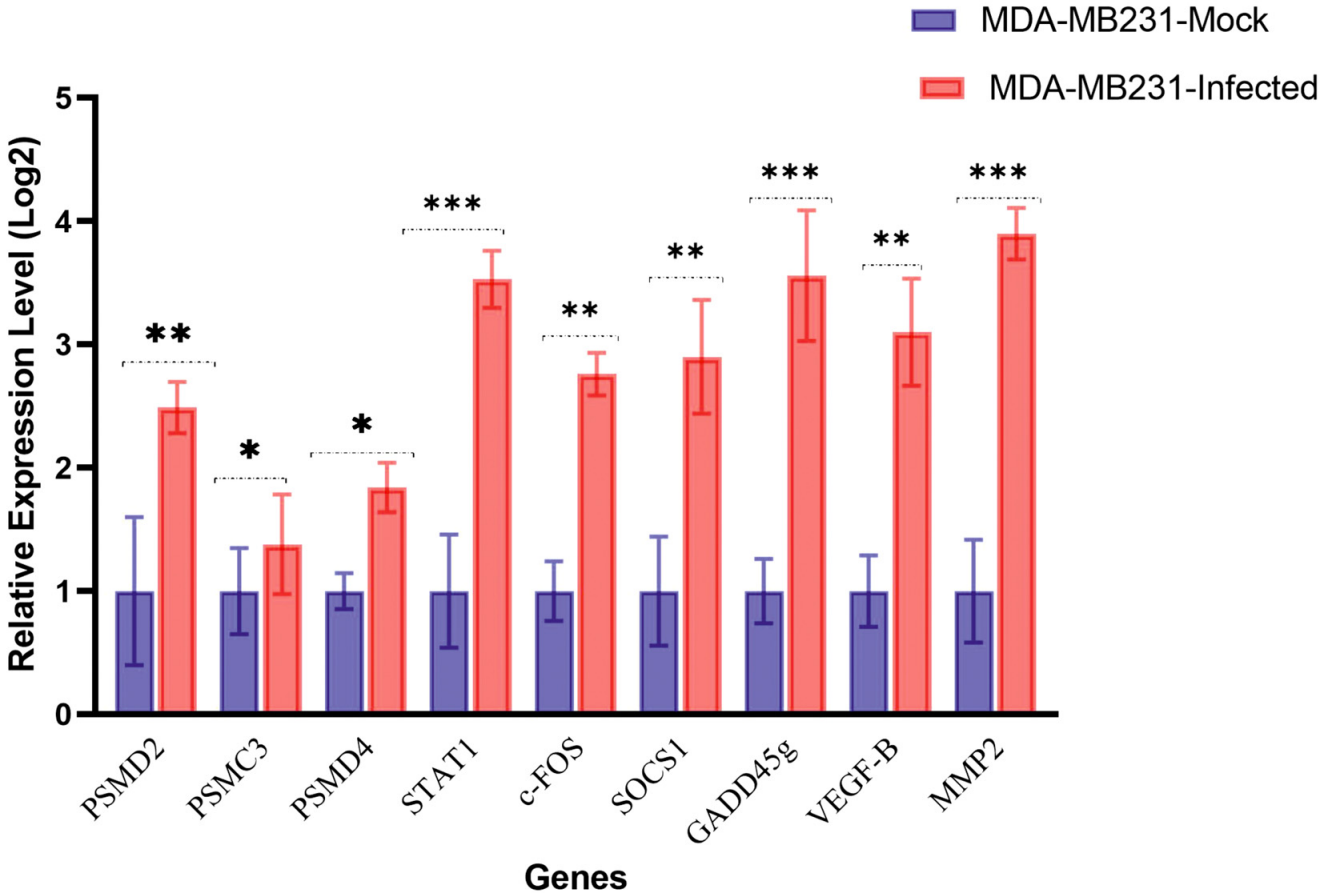
mRNA expression genes in MDA-MB-231 mock or infected with oHSV-1 by reverse transcription quantitative PCR. Gene expression levels were calculated based on the ΔΔCt relative quantification. Three biological replicates were performed. *p < 0.05, **p < 0.01, ***p < 0.001.

Based on the results obtained from the DEGs list and network analysis, three hub genes and six genes were meticulously selected from the up- and downregulated genes. These genes were chosen to measure their expression using the qRT-PCR method, including PSMD2, PSMD4, PSMC3, STAT1, c-Fos, SOCS1, GADD45g, VEGF-β, and matrix metalloproteinase 2 (MMP2). These selected genes are HSV-1-regulated genes involved in mRNA splicing, protein translation, transcriptional regulation, and cell survival. The qRT-PCR was performed to determine whether the mRNA expression levels of these genes in MDA-MB-231 cells infected with oHSV-1 and uninfected cells had been changed. The results, which revealed that all nine genes were differentially expressed and consistent with the microarray dataset results, further validated the accuracy of our findings (**Figure 7**).

## Discussion

In the present study, two microarray datasets were utilized to identify genes that were induced by oncolytic HSV-1 infection in human cancer cells. Through our analysis of these genes, key pathways that were altered after infection with the oncolytic virus were obtained. The results revealed that genes related to proteasomes were among the most prominent hub genes. Therefore, the PSMD2, PSMD4, and PSMC3 genes were selected from this pathway. Several genes with high expression in each dataset, which have important roles in cell signaling pathways linked to virus replication, were also selected. Ultimately, nine genes, including STAT1, c-Fos, SOCS1, GADD45g, VEGF-β, and MMP2, were chosen for the next phase of the experiment. In the final stage, the breast cancer cell line was infected with an oncolytic virus, and the expression levels of the selected genes were measured using qRT-PCR.

In our study, KEGG pathway enrichment data revealed a significant association of the differentially expressed genes with the proteasome pathway. Li et al. explained a combination therapy of an oHSV-1 and a proteasome inhibitor used on colorectal cancer cells. This combined treatment ultimately led to inhibiting the growth of tumor cells. Proteasome inhibitors, such as bortezomib, can directly bind to the 20S core active site in cells and reversibly inhibit the activity of the 26S proteasome. This action causes IκB (inhibitor of nuclear factor kappa B) aggregation, prevents NF-κB release, inhibits NF-κB activation, and inhibits tumor growth. Disrupting the protein degradation pathway causes disturbance in the metabolism of several proteins, leading to the activation of apoptotic pathways and inducing the apoptosis of tumor cells. These results suggest that incorporating proteasome inhibitors (in the form of either genetic material or protein) and oHSV-1 could be a promising strategy for developing future generations of oHSV-1 viruses (14, 15).

In the present investigation, we noted a rise in SOCS1 expression in breast cancer, a finding consistent with Mahller’s research on MPNST cells. Mahller et al. indicated that SOCS1 upregulation is not detectable in virus-insensitive cell lines. Hence, this gene could function as a surrogate predictor of oHSV sensitivity (5). Moreover, SOCS1 appears to have a crucial part in oHSV-1 replication. This suggests that SOCS1 expression, when introduced as a transgene in an oncolytic HSV-1 vector, may facilitate viral replication and oncolysis in cells that typically resist viral infection (16, 17).

Our research demonstrated that STAT1 expression increased in breast cancer cells infected with oHSV-1, consistent with Mahller’s study (5). Increased susceptibility of cancer cells to viruses is a critical prerequisite for the efficacy of oncolytic virotherapy. A key factor contributing to this phenotype is the impairment of innate antiviral defenses, which is linked to the dysfunction of type 1 interferons (IFNs). This dysfunction allows uncontrolled viral replication within cancer cells (18, 19). During HSV-1 infection, interferons and other cytokines can activate STAT1, leading to the expression of antiviral genes and preventing virus replication. Studies have shown that the insensitivity of cancer cells to specific viruses is associated with impaired IFN response, and JAK/STAT inhibitors can overcome this resistance to viral therapy (13, 20). The use of JAK/STAT inhibitors can enhance the efficiency of oncolytic viruses in resistant tumor cells. Additionally, Patel and colleagues employed the JAK/STAT inhibitor, ruxolitinib, in combination with VSV-IFNβ, and they found that inhibition of JAK/STAT signaling improved VSV-IFNβ therapy for lung cancer (21).

Our study revealed an increase in the expression of the c-Fos gene, as previously reported by Mahller et al. (5). The FOS family consists of four members: FOS, FOSB, FOSL1, and FOSL2. These leucine zipper proteins can dimerize with proteins of the JUN family, forming the transcription factor complex AP-1, which regulates cell proliferation, differentiation, and transformation (22). FOS proteins have also been implicated in apoptotic cell death. In immune responses, when specific antigens are presented by MHC molecules and recognized by T-cell receptors, transcription factors such as NFκB1, NFATC1, c-Jun, and c-Fos are activated. c-Fos activation leads to the production of various cytokines and chemokines, including IFNγ, T-bet, TNF, GM-CSF, IL-2, IL-4, IL-5, IL-10, and IL-13 (23, 24). In the case of HSV-1 infection, c-Fos expression can be triggered by viral proteins or cellular stress responses, as demonstrated by Hu et al. (10). Therefore, the unique properties of c-Fos may provide a promising therapeutic approach for targeting and destroying cancer cells through the design of an oncolytic HSV-1 virus. However, further research and preclinical studies would be necessary to evaluate the efficacy and safety of c-Fos in developing novel oncolytic virotherapy for cancer treatment.

We observed that VEGF-β expression was increased in breast cancer cells infected with oHSV-1, as reported by Kurozumi et al. (25). VEGF is critical in the signaling pathways that regulate angiogenesis, tumor growth, and metastasis. It is known to promote blood vessel formation during viral infections to aid in spreading the virus within the host (26, 27). The upregulation of VEGF-β during HSV-1 infection may contribute to the forming of new blood vessels within the tumor microenvironment, providing a nutrient-rich environment for viral replication and tumor progression (28, 29). Monoclonal antibodies against VEGF are widely used in clinical oncology due to their high expression in many cancers. This pathway can be targeted by oncolytic viruses that express angiogenesis inhibitors (VEGI), as mentioned in the study of Tysome et al. Targeting the VEGF pathway has been effective in animal models and shows promise for translation to clinical studies in the future (30, 31).

MMP2 is an enzyme involved in remodeling the extracellular matrix, tissue repair, and angiogenesis (32). Previous studies have shown that HSV-1 infection can induce the upregulation of MMP2 expression, leading to increased extracellular matrix degradation and tissue remodeling (33, 34). Ramos et al. have shown that MMP2 is a reliable predictor of tumor progression and metastasis (35).

Our results indicated that GADD45g expression was increased in breast cancer cells infected with oHSV-1. The GADD45g gene, also known as growth arrest and DNA damage-inducible gene 45 gamma, plays a crucial role in regulating various intracellular processes, including cell cycle arrest, DNA repair, and apoptosis (36, 37). Ravirala and colleagues have shown that the expression of GADD45g was upregulated during oHSV-1 infection, which is desirable for optimal virus replication (38). Additionally, GADD45g activation leads to the activation of the p38 MAPK pathway, which is crucial in regulating inflammatory and stress responses (39). Furthermore, GADD45g has been shown to modulate the expression of pro-apoptotic and anti-apoptotic genes, thereby influencing the balance between cell survival and cell death during HSV-1 infection (36, 40). Overall, the relationship between the GADD45g gene and intracellular processes during oncolytic HSV-1 infection highlights the complex interplay between host antiviral responses and viral replication strategies (41). Further research is needed to elucidate the precise mechanisms by which GADD45g contributes to the antiviral response against HSV-1 and its potential implications for oncolytic virotherapy.

## Conclusion

The efficacy of oncolytic viral therapy is contingent upon the target tissue. Therefore, it is imperative to comprehend the attributes of cancer cells, their microenvironment, and cell signaling pathways to devise therapeutic approaches. Further exploration into the role of genes triggered by oHSV-1 during viral replication will facilitate the formulation of rational strategies for creating the next generation of oncolytic viruses. Our study revealed that genes with altered expression in tumor cells infected with the oHSV-1 virus, such as GADD45g and c-Fos, can affect the function of the virus. In future research, scientists could gain a deeper understanding of the relationship between virus infection and tumor cell alterations by closely examining the transcriptome of cancer cells and utilizing this information to design viruses with maximum efficiency and safety.

## Data Availability

The original contributions presented in the study are included in the article/**Supplementary Material**. Further inquiries can be directed to the corresponding author.
