# Supplementary material for: Rational strategies for designing next-generation oncolytic viruses based on transcriptome analysis of tumor cells infected with oncolytic herpes simplex virus-1
Source: Front Oncol. 2025 Jan 9;14:1469511. doi: 10.3389/fonc.2024.1469511 (PMC11754274; doi:10.3389/fonc.2024.1469511)
Supplement: Supplementary file 1 [file Table1.docx]

Table S1: list of primer

| Name | sequence | Product size (bp) |
| --- | --- | --- |
| STAT1-F | ATGCACCCTGAAGAAGCTGT | 176bp |
| STAT1-R | CAGGACTGCAGAGAGGGAAC |  |
| FOS-F | GATGTAGCAAAACGCATGGA | 173bp |
| FOS-R | TCCAGCACCAGGTTAATTCC |  |
| SOCS1-F | CTGGGATGCCGTGTTATTTT | 252bp |
| SOCS1-R | TAGGAGGTGCGAGTTCAGGT |  |
| MMP2 -F | ACTGCTGGCTGCCTTAGAAC | 183bp |
| MMP2 -R | GTGAACAGGGGAACCATCAC |  |
| VEGFB-F | CCCTTGACTGTGGAGCTCAT | 242bp |
| VEGFB-R | GGCTTCACAGCACTGTCCTT |  |
| GADD45g-F | CGTCTACGAGTCAGCCAAAGTC | 146bp |
| GADD45g-R | CGATGTCGTTCTCGCAGCAGAA |  |
| PSMD2-F | GCCTCACCCAGATTGACAAG | 68bp |
| PSMD2-R | GGCAAGAAGAGCTCCTGACTTA |  |
| PSMD2-F | CTGGACATCATGGAGCCCAA | 266bp |
| PSMD2-R | CCACCATCCACATCCCACAG |  |
| PSMC3-F | TGAAGAGTGAAGTGTTGAGAG | 144bp |
| PSMC3-R | CATTAGGATCAACATCCAGGA |  |
| PSMD4-F | AGGTGGCAAGATGGTGTTGG | 170bp |
| PSMD4-R | GTGTGATAAGGCCCACGTTG |  |
| GAPDH-F | GAAGGTGAAGGTCGGAGT | 226bp |
| GAPDH-R | GAAGATGGTGATGGGATTTC |  |

Table S2: Differential Expression Genes (DEGs) of GSE8717 (the top 30 significant gene)

|  | Gene Symbol | P.Value | adj.P.Val | LogFC |
| --- | --- | --- | --- | --- |
| 1 | EXT2 | 3.50E-12 | 2.74E-08 | -0.448 |
| 2 | FAM134A | 2.19E-11 | 1.18E-07 | -0.340 |
| 3 | ADIPOR2 | 2.51E-11 | 1.18E-07 | -0.437 |
| 4 | ATRAID | 5.68E-11 | 2.15E-07 | -0.402 |
| 5 | TMEM248 | 6.39E-11 | 2.15E-07 | -0.526 |
| 6 | ZFP36 | 7.36E-11 | 2.16E-07 | 0.408 |
| 7 | RALY | 1.04E-10 | 2.71E-07 | -0.394 |
| 8 | ERI3 | 1.23E-10 | 2.89E-07 | -0.332 |
| 9 | TMEM184B | 1.65E-10 | 3.52E-07 | -0.379 |
| 10 | MBTPS1 | 1.89E-10 | 3.61E-07 | -0.198 |
| 11 | ARPC1A | 2.38E-10 | 3.61E-07 | -0.336 |
| 12 | MAPK9 | 2.47E-10 | 3.61E-07 | -0.340 |
| 13 | TSPAN4 | 2.49E-10 | 3.61E-07 | -0.421 |
| 14 | BCL2L11 | 2.57E-10 | 3.61E-07 | 0.266 |
| 15 | SERTAD1 | 2.71E-10 | 3.61E-07 | 0.298 |
| 16 | LINC00094 | 2.91E-10 | 3.61E-07 | -0.355 |
| 17 | PDXK | 3.02E-10 | 3.61E-07 | -0.213 |
| 18 | ERGIC1 | 3.07E-10 | 3.61E-07 | -0.261 |
| 19 | GOLM1 | 3.38E-10 | 3.79E-07 | -0.193 |
| 20 | CLIC3 | 4.62E-10 | 4.94E-07 | 0.284 |
| 21 | GBA /// GBAP1 | 5.11E-10 | 5.23E-07 | -0.327 |
| 22 | CENPB | 5.70E-10 | 5.58E-07 | -0.464 |
| 23 | SSRP1 | 6.88E-10 | 6.42E-07 | -0.417 |
| 24 | PDIA6 | 7.11E-10 | 6.42E-07 | -0.386 |
| 25 | SDC1 | 7.36E-10 | 6.42E-07 | -0.427 |
| 26 | ARC | 8.13E-10 | 6.83E-07 | 0.868 |
| 27 | PAM16 | 8.87E-10 | 6.98E-07 | -0.246 |
| 28 | GTF3C2 | 8.90E-10 | 6.98E-07 | -0.307 |
| 29 | MIR612 /// NEAT1 | 1.13E-09 | 8.18E-07 | 0.356 |
| 30 | GADD45B | 1.14E-09 | 8.18E-07 | 0.410 |

**Table S3:** Differential Expression Genes (DEGs) of GSE162643 (the top 30 significant gene)

|  | Gene Symbol | P.Value | adj.P.Val | LogFC |
| --- | --- | --- | --- | --- |
| 1 | COX1 | 2.24E-302 | 4.94E-298 | -37.168 |
| 2 | COX3 | 8.94E-252 | 9.86E-248 | -33.891 |
| 3 | TUG1 | 8.93E-234 | 6.57E-230 | -32.647 |
| 4 | SPDYE1 | 2.02E-208 | 1.12E-204 | 30.808 |
| 5 | ND4 | 1.08E-189 | 4.76E-186 | -29.377 |
| 6 | COX2 | 1.42E-186 | 5.23E-183 | -29.132 |
| 7 | ND4L | 1.80E-179 | 5.68E-176 | -28.566 |
| 8 | ATP6 | 7.31E-179 | 2.01E-175 | -28.517 |
| 9 | ND1 | 7.36E-174 | 1.80E-170 | -28.110 |
| 10 | ND5 | 4.31E-173 | 9.51E-170 | -28.047 |
| 11 | TRNS1 | 2.94E-162 | 5.89E-159 | -27.145 |
| 12 | ND6 | 1.42E-159 | 2.61E-156 | -26.916 |
| 13 | MIR100HG | 4.76E-151 | 8.08E-148 | -26.178 |
| 14 | MIR12136 | 5.01E-147 | 7.89E-144 | -25.822 |
| 15 | ATP8 | 3.59E-145 | 5.28E-142 | -25.656 |
| 16 | NORAD | 7.99E-141 | 1.10E-137 | -25.264 |
| 17 | LOC107984026 | 9.24E-140 | 1.20E-136 | 25.167 |
| 18 | CYTB | 3.48E-139 | 4.26E-136 | -25.114 |
| 19 | TRNE | 2.86E-137 | 3.32E-134 | -24.938 |
| 20 | FGD5-AS1 | 2.83E-133 | 3.12E-130 | -24.567 |
| 21 | CHRNB2 | 1.14E-130 | 1.20E-127 | -24.322 |
| 22 | SNHG29 | 1.65E-129 | 1.65E-126 | -24.212 |
| 23 | TRNY | 9.59E-128 | 9.19E-125 | -24.044 |
| 24 | SPDYE18 | 6.34E-124 | 5.83E-121 | 23.676 |
| 25 | SRCAP | 3.40E-118 | 3.00E-115 | 23.113 |
| 26 | TRNC | 7.66E-115 | 6.50E-112 | -22.778 |
| 27 | WAC-AS1 | 7.14E-114 | 5.83E-111 | -22.679 |
| 28 | TRNN | 1.07E-111 | 8.44E-109 | -22.458 |
| 29 | LOC101927556 | 1.11E-107 | 8.41E-105 | -22.043 |
| 30 | HCG18 | 2.50E-107 | 1.84E-104 | -22.006 |
